# Supplementary material for: Time-series transcriptome analysis identified differentially expressed genes in broiler chicken infected with mixed Eimeria species
Source: Front Genet. 2022 Aug 8;13:886781. doi: 10.3389/fgene.2022.886781 (PMC9393255; doi:10.3389/fgene.2022.886781)
Supplement: Supplementary file 2 [file DataSheet1.ZIP › 7dpi_GO.Gsea.1625106495923/GOBP_CYCLIC_NUCLEOTIDE_METABOLIC_PROCESS.html]

Details for gene set GOBP\_CYCLIC\_NUCLEOTIDE\_METABOLIC\_PROCESS[GSEA]

|  || Dataset | TMM\_7dpi\_gct\_format\_7dpi\_gct\_format.Class\_4dpi.cls #PC\_versus\_NC.Class\_4dpi.cls #PC\_versus\_NC\_repos |
| Phenotype | Class\_4dpi.cls#PC\_versus\_NC\_repos |
| Upregulated in class | 0 |
| GeneSet | GOBP\_CYCLIC\_NUCLEOTIDE\_METABOLIC\_PROCESS |
| Enrichment Score (ES) | -0.6356529 |
| Normalized Enrichment Score (NES) | -1.8409855 |
| Nominal p-value | 4.9321825E-4 |
| FDR q-value | 0.09913192 |
| FWER p-Value | 0.805 |
Table: GSEA Results Summary

  

Fig 1: Enrichment plot: GOBP\_CYCLIC\_NUCLEOTIDE\_METABOLIC\_PROCESS      
 Profile of the Running ES Score & Positions of GeneSet Members on the Rank Ordered List

  

| SYMBOL | TITLE | RANK IN GENE LIST | RANK METRIC SCORE | RUNNING ES | CORE ENRICHMENT || 1 | EPHA2 | na | 752 | 0.610 | -0.0217 | No |
| 2 | PDE4C | na | 1374 | 0.411 | -0.0459 | No |
| 3 | PDE9A | na | 1448 | 0.395 | -0.0254 | No |
| 4 | ADCY9 | na | 2142 | 0.246 | -0.0667 | No |
| 5 | PDE4B | na | 3069 | 0.117 | -0.1361 | No |
| 6 | PDE10A | na | 3309 | 0.086 | -0.1503 | No |
| 7 | CNP | na | 3358 | 0.079 | -0.1490 | No |
| 8 | NPR2 | na | 3645 | 0.048 | -0.1696 | No |
| 9 | ADCY3 | na | 5416 | -0.141 | -0.3078 | No |
| 10 | ADCY6 | na | 5876 | -0.188 | -0.3335 | No |
| 11 | AMPD2 | na | 7716 | -0.386 | -0.4609 | No |
| 12 | PDE7A | na | 9531 | -0.636 | -0.5695 | No |
| 13 | GUCY1A2 | na | 10325 | -0.775 | -0.5835 | Yes |
| 14 | PDE7B | na | 10356 | -0.781 | -0.5335 | Yes |
| 15 | PTHLH | na | 10585 | -0.829 | -0.4968 | Yes |
| 16 | PDE8A | na | 10596 | -0.833 | -0.4417 | Yes |
| 17 | ADCY8 | na | 10620 | -0.838 | -0.3872 | Yes |
| 18 | GUCY2C | na | 10748 | -0.866 | -0.3396 | Yes |
| 19 | PDE4D | na | 11119 | -0.969 | -0.3053 | Yes |
| 20 | RORA | na | 11305 | -1.039 | -0.2509 | Yes |
| 21 | PDE5A | na | 11464 | -1.102 | -0.1900 | Yes |
| 22 | ADCY2 | na | 11469 | -1.105 | -0.1161 | Yes |
| 23 | PDE1A | na | 11477 | -1.107 | -0.0422 | Yes |
| 24 | ADCY7 | na | 11750 | -1.288 | 0.0217 | Yes |
Table: GSEA details [plain text format]

  

Fig 2: GOBP\_CYCLIC\_NUCLEOTIDE\_METABOLIC\_PROCESS      
 Blue-Pink O' Gram in the Space of the Analyzed GeneSet

  

Fig 3: GOBP\_CYCLIC\_NUCLEOTIDE\_METABOLIC\_PROCESS: Random ES distribution      
 Gene set null distribution of ES for **GOBP\_CYCLIC\_NUCLEOTIDE\_METABOLIC\_PROCESS**
